# Interplay between TRα1 and Wnt signaling: A dangerous liaison

**DOI:** 10.18632/oncotarget.25807

**Published:** 2018-08-10

**Authors:** Sheue-Yann Cheng

**Affiliations:** Laboratory of Molecular Biology, Center for Cancer Research, National Cancer Institute, Bethesda, Maryland, USA

**Keywords:** thyroid hormone receptor isoforms, tumor suppressor, oncogene, Wnt signaling, TRβ mutations

In the past decades, great strides forward have been made in the understanding of genomic actions of thyroid hormone nuclear receptors (TRs). One aspect of TR actions that has received relatively little attention until recently is the role of TRs in carcinogenesis. There are two major thyroid hormone (T3) binding TRs—TRα1 and TRβ1. They share high sequence homology in the DNA and T3 binding domains, but they totally differ in the amino terminal A/B domain in the length and amino acid sequences. Increasing evidence from several groups of investigators has clearly demonstrated that TRβ1 could function as a tumor suppressor in cultured cells *in vitro* and in xenograft models *in vivo* [1-3]. Less explored has been whether TRα1 plays a role in carcinogenesis.

However, the article by Uchuya-Castillo *et al* in this issue reports the detection of increased expression of TRα1 in colorectal tumors of patient cohorts. Importantly, the *THRA* gene expression levels were significantly and directly correlated with Wnt activity. Previously, the same group reported that the ectopic expression of TRα1 in the intestine epithelium of *Apc^+/1638N^* mice (vil-TRα1/*Apc^+/1638N^* mice) accelerated tumorigenesis with more aggressive tumor phenotypes [4]. Uchuya-Castillo *et al* have now further demonstrated that cellular TRα1 levels regulate Wnt activity to affect colon cancer cell proliferation and migration. The differential transcriptomic profiles in the adenocarcinomas of *Apc^+/1638N^* mice and vil-TRα1/*Apc^+/1638N^* mice further support the functional link of TRα1 to Wnt signaling. They found that increased expression of TRα1 was accompanied by decreased levels of several cellular inhibitors of Wnt signaling. Such inverse correlation found in the mouse models was also demonstrated in colorectal tumors of patient cohorts to account for how the elevated TRα1 led to the activation of Wnt signaling, thereby establishing the potential oncogenic role of TRα1 in the intestine epithelium.

The outcome of the present work highlights the contrasting functions of TR isoforms in tumorigenesis. Many lines of evidence have indicated that TRβ1 functions as a tumor suppressor. Loss of the *THRB* gene by chromosome deletion, as well as silencing in the expression of the *THRB* gene by promoter hypermethylation, has been reported in many human cancers. The tumor suppressing functions of TRβ1 have been demonstrated in many tumor cell lines derived from many cancers including thyroid cancer, breast cancer, and hepatocellular carcinoma. The compelling *in vivo* evidence to support the tumor suppressor role of TRβ1 came from a mouse model in which the loss of tumor suppressor functions by mutations causes spontaneous development of follicular thyroid cancer (*Thrb^PV/PV^* mouse) [5]. However, it is important to point out that the mutated TRβPV, identified in a patient with resistance to thyroid hormone (RTHβ), does not act alone to initiate thyroid carcinogenesis. In the *Thrb^PV/PV^* mouse, TRβPV collaborates and synergizes with other activated pathways such as thyroid stimulating hormone (TSH) [6] and PI3K-AKT signaling [7] to induce the thyroid cancer phenotype [1]. In a similar fashion, TRα1 alone, as shown in vil-TRα1 mice [4], does not initiate cancer development in the intestine epithelium. As shown in vil-TRα1/*Apc^+/1638N^* mice, synergy of elevated TRα1 with Wnt signaling leads to tumorigenesis. While TR isoforms exhibit contrasting roles in tumorigenesis, the common theme is that both isoforms, via diverse and extensive cross-talks with other cellular regulators, synergize with tumor promoters and/or attenuate tumor suppressor functions, leading to tumorigenesis.

Earlier, mice with single or double TR isoform knockout have clearly revealed that TR isoforms exhibit redundant functions as well as isoform-specific actions. That TRα1 and TRβ1 exhibit contrasting molecular actions in tumorigenesis has further expanded the scope of isoform-dependent actions *in vivo*, which would compel the need to address a long-standing important biological question as to why there are TR isoform-dependent actions *in vivo*. At present, little is known about the molecular basis underlying the isoform-dependent actions*.* In view of the findings that both TR isoforms necessitate the collaborations and synergy with other regulators critical in cellular functions to affect tumorigenesis, it would be reasonable to speculate that the divergent amino-terminal A/B domains in the two isoforms could play an important role in dictating the isoform-dependent functions. The three-dimensional structure for each TR isoform, while currently unknown, could form distinct interacting surfaces to recruit different transcription factors and/or enhancers on chromatin to affect gene transcription. The tissue-dependent expression of TR isoforms could further expand the combinatory networks, leading to the manifestation of TR isoform actions in a cellular-context-dependent manner. However, this critical question requires additional studies.

The findings reported by Uchuya-Castillo *et al* in this issue should prompt further studies to address several critical questions. 1). In patients with colorectal cancer as well as in vil-TRα1/*Apc^+/1638N^* mice, the increased TRα1 level is the key to activate Wnt signaling to induce tumorigenesis. Thus, it would be important to identify the cellular factors that could elevate TRα1 levels in intestine epithelium, ultimately leading to colorectal cancer. 2). Is there a defined threshold in the ‘elevated” TRα1 that could impact the development of colorectal cancer? 3). Can the elevated expression of TRα1 in other tissues in which a known oncogenic mutation is expressed promote cancer development? 4). Wnt is expressed in many tissues initially during development and later during growth. Can elevated TRα1 synergize with Wnt signaling in other tissues to impact tumorigenesis? These questions together with others yet to be defined could help to further our understanding the oncogenic actions of TRα1. The uncovering of the oncogenic actions of the elevated TRα1 in the intestine epithelium reported by Uchuya-Castillo *et al* is a beginning to spur additional studies.
